# In the Night Time

**Published:** 1886-12-04

**Authors:** Helen Milman


### IN THE NIGHT TIME.

"'Tis in the hour of darkness, Lord, I feel
That pain is hard to bear,
Before the day-light dawns to bid us know
That sunshine still is there,
When all is still, sweet sleep forsakes, and I
Lie longing for Thy summons to the sky.

"I cannot rest, Lord, art Thou sleeping on—
While I am here in pain?
Who bidst me bear this burden patiently,
And bidst me count it gain,
Give me, oh! give me strength to bravely wait
Till Thou shalt greet me at the Golden Gate.

"I once thought well that I had power to bear,
Ready indeed to stand
And clasp the cross of suff'ring, for Thy sake,
A gift from Thine own hand!
But now I cry to Thee! To Heav'n above!
While owning that the deed was done in Love."

"I am not sleeping, weary heart, but here
I watch beside thy bed,
I know thy pain, and long to call thee Home
To endless peace instead.
Still for My work *I need thee*, so 'tis best
One with Me, bravely bear; 'tis Mine the rest.'"

Helen Milman.

